# Research progress of gut microbiota and its metabolites in polycystic ovary syndrome

**DOI:** 10.3389/fendo.2025.1700191

**Published:** 2025-12-17

**Authors:** Shiyi Qin, Shimeng Guo, Xinmiao Tan, Ke Li, Jiayu Huang

**Affiliations:** 1 Chongqing Key Laboratory of Human Embryo Engineering and Precision Medicine, Center for Reproductive Medicine, Chongqing Health Center for Women and Children, Women and Children’s Hospital of Chongqing Medical University, Chongqing, China; 2 Department of Orthopedics Surgery, The First Affiliated Hospital of Chongqing Medical University, Chongqing, China; 3 Chongqing Municipal Health Commission Key Laboratory of Musculoskeletal Regeneration and Translational Medicine, Chongqing, China; 4 Orthopedic Laboratory of Chongqing Medical University, Chongqing, China; 5 Reproductive Medicine Center, The First Affiliated Hospital of Chongqing Medical University, Chongqing, China

**Keywords:** polycystic ovary syndrome, gut microbiota, short chain fatty acids, lipopolysaccharide, bile acids, trimethylamine N-oxide, branched chain amino acids, intestinal fungus

## Abstract

Polycystic ovary syndrome (PCOS) ranks among the most widespread endocrine and metabolic conditions affecting women of childbearing age, but its specific pathogenesis remains unknown. More and more evidence indicates that PCOS may be a complex polymorphic disease, influenced by epigenetic and environmental factors, including diet and lifestyle. This review focuses on the role of the gut microbiota and its metabolites in PCOS, a topic that has gained significant attention recently due to the established link between the gut microbiome and metabolic disorders.

## 1 Introduction

Polycystic ovary syndrome (PCOS) is one of the most common endocrine, metabolic, and reproductive disorders in women of reproductive age, with a reported prevalence of 4%-20% (1, 2). The wide range reflects heterogeneity in diagnostic criteria, study populations, designs, and handling of undiagnosed cases. Current clinical diagnosis mainly follows the criteria proposed at the Rotterdam Conference in the Netherlands in 2003. Diagnostic confirmation is achieved if any two of the following three criteria are fulfilled: 1. Oligo-ovulation or anovulation; 2. Polycystic ovarian changes; 3. Hyperandrogenism (HA), excluding other endocrine abnormalities causing it (3). PCOS is closely associated with obesity, insulin resistance (IR), chronic inflammation, type 2 diabetes, and cardiovascular diseases (4, 5). However, its specific pathogenesis has not been fully elucidated. More and more evidence suggests that PCOS may be a complex polygenic disease, and its onset is not only related to epigenetic factors but also environmental factors (6, 7).

The gut microbiome, referred to as “the second genome of human beings,” comprises roughly 100 trillion microorganisms inhabiting the human gastrointestinal system, establishing an interdependent symbiotic relationship with the host (8, 9). It plays a vital role in fundamental physiological functions, including neuroendocrine-immune modulation, digestion, medication metabolism, and endotoxin clearance (10, 11). In recent years, the relationship between gut microbiota and PCOS has become a central topic of investigation. PCOS patients commonly exhibit alterations in their gut microbiota composition, resulting in impaired glucose metabolism, dysregulated lipid metabolism, and disturbances in sex hormone balance (12, 13). This article reviews the mechanisms by which gut microbiota and their metabolites contribute to the development of PCOS.

## 2 Gut microbiota diversity and compositional changes in PCOS

### 2.1 Alpha and beta diversity of the gut microbiota in PCOS

Robust microbial diversity is fundamental to the gut microbiota’s resilience against stressors and serves as a primary indicator of overall health. Conversely, a reduction in this diversity, often driven by the proliferation of pathogenic microorganisms, signals a compromised physiological state (14). In PCOS patients, the gut microbiota often exhibits altered diversity. Specifically, α-diversity reflects the richness and variety of species within a specific community, while β-diversity measures the similarity or difference between distinct samples or groups (12).

Numerous clinical studies have indicated that the α-diversity of gut microbiota is significantly reduced in PCOS patients. Commonly used α-diversity indices in current research, such as Shannon index, Chao1 index, Simpson index, and observed operational taxonomic units (OTUs), are generally lower in the PCOS group compared to healthy controls (15–21). However, some clinical studies failed to observe significant differences in α-diversity between PCOS patients and healthy controls (22–25). Notably, Dong et al. (26) conducted a subgroup analysis of PCOS patients, revealing that significant differences in the Shannon index persisted between specific PCOS subgroups and the control group, when stratified by homeostasis model assessment of insulin resistance (HOMA-IR) or body mass index (BMI). This suggests that an overall analysis may obscure α-diversity alterations within distinct subgroups. In animal model studies, both Torres’s (27) and Han’s (28) studies observed a decrease in the Faith’s phylogenetic diversity (PD) index in the PCOS model group. Conversely, studies by Zheng (29), Zheng (30), Rodriguez Paris (31), Yu (32), and Esfandiarinezhad (33) showed that α-diversity indices such as Chao1, Shannon, Simpson, abundance-based coverage estimator(ACE), and species evenness in the PCOS animal model group were not significantly different from the control group. However, Li et al. (34) observed in their animal study that the amplicon sequence variants (ASVs) count, Shannon, and Simpson indices in the PCOS model group were, on the contrary, increased.

Regarding β-diversity, most current studies are analyzed using methods such as principal coordinate analysis (PCoA) and permutational multivariate analysis of variance (PERMANOVA), primarily based on distances like UniFrac and Bray-Curtis. In clinical studies, some have detected significant differences in gut microbiota structure between PCOS patients and healthy controls (15, 18–22, 25, 35). However, several other studies failed to observe significant differences in β-diversity (16, 17, 23, 24, 26, 36). Among these studies, Chu (36), Dong (26), and He (23) further performed subgroup analyses of PCOS patients stratified by BMI or IR. Even after such stratification, they still failed to detect significant differences in gut microbiota structure between these PCOS subgroups and the control group. In animal model studies, most investigations identified significant differences in gut microbiota β-diversity between PCOS model groups and control groups (21, 27, 29, 31–34), with only a few studies reporting no significant differences (28, 30, 37). Among these, Zheng (30) and Pieczyńska-Zając (37) noted that significant changes were observed in the high-fat PCOS subgroup.

It can be seen that the current research results on gut microbiota diversity in PCOS exhibit a certain degree of heterogeneity, both in clinical cohorts and animal models. However, the overall trend indicates that PCOS may be associated with changes in gut microbiota diversity. Han et al. (38) analyzed and found that the α-diversity of animal models showed higher stability. They proposed that the discrepancy in results between PCOS clinical studies and animal studies might be attributed to the strict control of environmental factors (such as diet and genetics) in animal experiments, which minimizes external confounding variables. In contrast, most human studies do not control or standardize patients’ diets, leading to fluctuations in the detected results of gut microbiota composition and diversity. (**Table 1**).

**Table 1:** Alpha and beta diversity of the gut microbiota in PCOS.

Study type	Author/Year	Sample groups	Sequencing method	α-diversity change	β-diversity change
Clinical Study	Torres 2018 (15)	PCOS (n=73)/control (n=48)	2nd-gen 16S rRNA sequencing (V4)	Decreased: Observed SVs, Faith’s PD, Shannon, indices	Significant difference: UniFrac distance (PCoA, PERMANOVA)
Clinical Study	Qi 2019 (22)	PCOS (n=50)/control (n=43)	Whole-genome shotgun sequencing	No significant change: Shannon index	Significant difference: Bray-Curtis distance(PERMANOVA), PLS-DA
Clinical Study	Zhang 2019 (35)	PCOS (n=38)/control (n=26)	2nd-gen 16S rDNA sequencing (V3-V4), deep shotgun metagenomic sequencing	-	Significant difference: UniFrac distance(PCoA)
Clinical Study	Chu 2020 (36)	PCOS (n=14)/control (n=14)	Shotgun metagenomic sequencing	-	No significant difference: PCA, ANOVA
Clinical Study	Zhou 2020 (16)	Obese PCOS (n=18)/control (n=15)	2nd-gen 16S rRNA sequencing	Decreased: Sobs, Shannon index, Rank-Abundance curve	No significant difference: OTU distribution(PCoA)
Clinical Study	Dong 2021 (26)	PCOS (n=45)/control (n=37)	16S rDNA full-length assembly sequencing	No statistical significance: Shannon index	No significant difference: PLS-DA
Clinical Study	He 2021 (23)	PCOS-IR (n=14)/PCOS-NIR (n=12)/control (n=10)	2nd-gen 16S rDNA sequencing (V3-V4)	No significant change: Observed OTUs, Chao1, Simpson, Shannon indices	No significant difference: UniFrac distance(PCoA)
Clinical Study	Yang 2022 (17)	PCOS (n=32)/control (n=18)	Shotgun metagenomic sequencing	Decreased: Shannon, Simpson index	No significant difference: Bray-Curtis distance(PCoA)
Clinical Study	Yu 2022 (18)	PCOS (n=20)/control (n=20)	2nd-gen 16S rRNA sequencing (V3-V4)	Decreased: Chao1, Shannon index, ASV rank abundance curve	Significant difference: Bray-Curtis distance(PCoA, NMDS)
Clinical Study	Li 2022 (24)	PCOS (n=31)/control (n=27)	2nd-gen 16S rRNA sequencing (V3-V4)	No significant change: Shannon, Simpson, Chao, ACE indices	No significant difference: OTU distribution(PCoA, NMDS, Venn diagram)
Clinical Study	Wang 2023 (25)	PCOS (n=24)/control (n=24)	2nd-gen 16S rDNA sequencing	No significant change: Shannon, Chao1, ACE indices	Significant difference: UniFrac/Bray-Curtis distance(PCoA)
Clinical Study	Silva 2024 (19)	PCOS (n=21)/control (n=10)	2nd-gen 16S rRNA sequencing (V4)	Decreased: Observed taxa, ACE, Simpson, Shannon indices	Significant difference: Bray-Curtis distance(PCoA, ANOSIM)
Clinical Study	Chen 2024 (20)	PCOS (n=17)/control (n=17)	2nd-gen 16S rRNA sequencing (V3-V4)	Decreased: Observed OTUs, Chao1, Shannon, Simpson, pielou_e indices	Significant difference: UniFrac/Bray-Curtis distance(PCoA, ANOSIM)
Animal Study	Torres 2019 (27)	Letrozole-induced PCOS(n=8)/control(n=8)	2nd-gen 16S rRNA sequencing (V4)	Decreased: Faith PD index	Significant difference: UniFrac distance(PCoA, CAP, PERMANOVA)
Animal Study	Zheng 2021 (29)	DHT-induced PCOS(n=5)/control(n=5)	2nd-gen 16S rDNA sequencing (V3-V4)	No significant change: Chao1, Shannon, Simpson indices	Significant difference: UniFrac distance(PCoA, ANOSIM)
Animal Study	Han 2021 (28)	DHEA-induced PCOS(n=6)/control(n=6)	2nd-gen 16S rRNA sequencing (V3-V4)	Decreased: Faith PD index	No significant difference: UniFrac distance(PCoA)
Animal Study	Zheng 2021 (30)	Letrozole-induced PCOS(n=5)/control(n=5)	2nd-gen 16S rRNA sequencing (V3-V4)	No significant change: Chao1, ACE, Shannon, Simpson indices	No significant difference: Bray-Curtis distance(PCoA, ANOSIM)
Animal Study	Rodriguez Paris 2022 (31)	DHT-induced PCOS/control	2nd-gen 16S rRNA sequencing (V4)	No significant change: Chao1, Species evenness, Shannon index	Significant difference: Bray-Curtis distance(PCoA, PERMANOVA), CCA, RDA
Animal Study	Li 2023 (34)	Letrozole-induced PCOS(n=10)/control(n=10)	2nd-gen 16S rRNA sequencing (V3-V4)	Increased: ASVs count, Shannon, Simpson indices	Significant difference: Bray-Curtis distance(PCoA, PERMANOVA)
Animal Study	Yu 2024 (32)	DHEA-induced PCOS(n=10)/control(n=10)	2nd-gen 16S rRNA sequencing (V3-V4)	No significant change: Chao1, ACE, Shannon, Simpson indices	Significant difference: Bray-Curtis distance(PCoA)
Animal Study	Pieczynska-Zajac 2024 (37)	Letrozole-induced PCOS(n=8)/control(n=8)	2nd-gen 16S rRNA sequencing (V3-V4)	No significant change: Shannon, Simpson indices	No significant difference: Bray-Curtis distance(PCoA, PERMANOVA)
Animal Study	Esfandiarinezhad 2025 (33)	DHT-induced PCOS(n=21)/control(n=18)	3rd-gen 16S rRNA sequencing	No significant change: Shannon index	Significant difference: UniFrac distance(PCoA)
Clinical and Animal Study	Yang 2021 (21)	Clinical: PCOS (n=56)/control (n=31); Animal: Letrozole-induced PCOS(n=8)/control(n=9)	2nd-gen 16S rRNA sequencing (V4)	Decreased: Shannon, Chao1, Observed_otus, PD_whole_tree indices	Significant difference: UniFrac distance(PCoA)

2nd-gen 16S rRNA sequencing, second-generation 16S ribosomal ribonucleic acid sequencing; 3rd-gen 16S rRNA sequencing, third-generation 16S ribosomal ribonucleic acid sequencing; 16S rDNA sequencing, 16S ribosomal deoxyribonucleic acid sequencing; SVs, sequence variants; PCA, principal component analysis; PLS-DA, partial least squares discriminant analysis; ANOVA, analysis of variance; NMDS, non-metric multidimensional scaling; ANOSIM, analysis of similarities; CCA, canonical correspondence analysis; RDA, redundancy analysis; CAP, canonical analysis of principal coordinates; DHT, dihydrotestosterone; DHEA, dehydroepiandrosterone

### 2.2 Gut microbiota compositional changes in PCOS

The dysbiosis of the gut microbiome in patients with PCOS may not be characterized solely by a decrease in overall diversity, but rather by a more pronounced manifestation of specific compositional changes in certain taxa at the phylum, genus, or species level (36, 39).

In PCOS, a significant increase in the abundance of certain potentially pathogenic and pro-inflammatory bacterial groups is observed. Specifically, the phylum *Proteobacteria* and its constituent family *Enterobacteriaceae*, particularly the genera *Escherichia/Shigella*, exhibit elevated abundance. These opportunistic pathogens are known to secrete virulence factors, thereby exacerbating systemic inflammation and metabolic dysfunction (18, 35, 36). Furthermore, within the phylum *Bacteroidota*, the family *Bacteroidaceae*, especially species like *Bacteroides vulgatus* and *Bacteroides fragilis*, show increased abundance. These specific bacteria contribute to the exacerbation of IR, ovarian dysfunction, and systemic inflammation through mechanisms such as interfering with bile acids (BAs) metabolism, compromising the intestinal mucosal barrier, and degrading mucin (22, 36). Similarly, species within the genus *Prevotella*, including *Prevotella stercorea* and *Prevotella copri*, also demonstrate increased abundance. They are implicated in promoting intestinal inflammation, leading to systemic metabolic abnormalities, and influencing branched-chain amino acids (BCAAs) synthesis and host metabolism (26, 35, 40). Concurrently, there is a general reduction in the abundance of beneficial bacteria crucial for gut health and short chain fatty acids (SCFAs)-producing bacteria. The overall abundance of the phylum *Bacteroidota* is generally diminished, which may impair its role in regulating intestinal barrier function and maintaining metabolic health (20, 29). Within the phylum *Firmicutes*, important SCFAs-producing bacterial families, such as *Lachnospiraceae* and *Ruminococcaceae*, show significantly decreased abundance. This reduction leads to diminished production of SCFAs like butyrate, consequently weakening intestinal barrier function, anti-inflammatory effects, and metabolic regulatory capacity (20, 21, 35). *Firmicutes* and *Bacteroidota* are the two most dominant phyla in the human gut microbiota, and their relative abundance ratio (F/B ratio) is considered a critical indicator for assessing gut dysbiosis. An elevated F/B ratio is typically closely associated with host metabolic dysregulation, impaired intestinal barrier function, immune dysregulation, and the development and progression of various metabolic diseases, including obesity and IR (41–43) (**Table 2**).

**Table 2:** Gut microbiota compositional changes in PCOS.

Phylum	Abundance change & function	Family	Abundance change & function	Genus	Abundance change & function	Species	Abundance change & function
*Proteobacteria*	Increased abundance (18, 20): Contains opportunistic pathogens.	*Enterobacteriaceae*	Increased abundance (23, 36): Exacerbates inflammation and metabolic disorders.	*Escherichia/*Shigella	Increased abundance (35, 36): Opportunistic pathogens, secrete virulence factors.	*-*	-
		*Pseudomonadaceae*	Decreased abundance (44)	*Pseudomonas*	Decreased abundance (44): Affects hormone and lipid metabolism.	*-*	-
*Firmicutes*	Increased abundance (29, 44): Promotes calorie absorption and fat storage; Decreased abundance (20): Contains SCFAs producers.	*Lachnospiraceae*	Decreased abundance (20, 21, 35): SCFAs producers, affects gut barrier function and metabolic regulation.	*Faecalibacterium*	Decreased abundance (35, 36)	*Faecalibacterium prausnitzii*	Decreased abundance (26, 35, 36): SCFAs producer (butyrate), associated with anti-inflammation.
				*Roseburia*	Decreased abundance (17, 18): Anti-inflammatory and metabolic regulation.	*Roseburia intestinalis*, Roseburia hominis	Decreased abundance (17, 35): SCFAs producers.
		*Ruminococcaceae*	Decreased abundance (20, 21): SCFAs producers.	*Ruminococcus*	Decreased abundance (33); Increased abundance (20, 26)	*Ruminococcus gnavus*	Increased abundance (20, 26): Utilizes mucin glycans as an energy source, associated with inflammation.
		*Streptococcaceae*	Increased abundance (20, 23): Pro-inflammatory bacteria.	*-*	-	*-*	-
*Bacteroidota*	Decreased abundance (20, 29): Regulates gut barrier function, associated with metabolic disorders.	*Bacteroidaceae*	Increased abundance (22, 36)	*Bacteroides*	Increased abundance (22, 35): Interferes with bile acid metabolism, disrupts gut barrier; Decreased abundance (19, 25): SCFAs producers, reduces inflammation.	*Bacteroides vulgatus*	Increased abundance (22, 35): Encodes BSH genes, interferes with bile acid metabolism, exacerbates IR and ovarian dysfunction.
						*Bacteroides fragilis*	Increased abundance (26, 36): Degrades mucin, disrupts gut barrier, promotes inflammation.
		*Prevotellaceae*	Decreased abundance (23): Involved in digestion and metabolic maintenance.	*Prevotella*	Increased abundance (26): pro-inflammatory.	*Prevotella stercorea*	Increased abundance (26): Promotes gut inflammation and systemic metabolic abnormalities.
						*Prevotella copri*	Increased abundance (35): Regulates BCAAs synthesis, affecting host metabolism.

BSH, Bile Salt Hydrolase.

### 2.3 Changes in the gut microbiota of PCOS subtypes

#### 2.3.1 HA

HA is a core characteristic of the reproductive phenotype in PCOS. It not only precipitates clinical manifestations such as acne, hirsutism, and androgenetic alopecia, but also induces ovarian stromal hyperplasia, accelerates follicular atresia, and disrupts gonadal axis hormone regulation, thereby impairing follicular growth, development, and ovulation (45, 46). There is a close bidirectional regulatory relationship between gut microbiota and androgen levels. Han et al. (28) demonstrated in a rat model of PCOS that DHEA induces gut dysbiosis, significantly reducing genera like *Turicibacter* and *Clostridium sensu stricto*. Fecal microbiota transplantation (FMT) from these rats into pseudo-germ-free recipients then elevated serum testosterone (T) levels, establishing an “androgen-microbiota” vicious cycle. Compared with normally ovulating women, the gut microbiota structure of PCOS patients tends to be simplified, and this degree of simplification is positively correlated with androgen levels (35). Torres et al. (15) performed 16S rRNA gene sequencing on 73 PCOS patients and 48 healthy controls, revealing a significant reduction in α-diversity in PCOS patients, which negatively correlated with T levels. Furthermore, HA statistically impacted β-diversity, significantly altering the overall structural composition of the gut microbiota. Analyzing PCOS subtypes, Li et al. (47) found that the HA-PCOS group exhibited significantly lower Chao1 and Shannon indices compared to the non-HA PCOS control group, indicating reduced species richness and evenness in HA-PCOS patients. Cluster analysis further confirmed significant differences in community structure between the two groups, underscoring the strong association between the hyperandrogenic state and alterations in overall gut microbiota composition. The findings of the study by Yang et al. (17) indicate that certain enriched bacterial strains in PCOS patients can directly contribute to HA. For example, *Clostridium scindens* can convert glucocorticoids into androgens via specific metabolic pathways, thereby directly elevating serum androgen levels. Furthermore, the *Pseudomonas* sp. *M1 strain* exacerbates androgen excess by expressing sulfatase, which converts inactive dehydroepiandrosterone sulfate (DHEAS) into its active form, DHEA.

#### 2.3.2 IR

In addition to reproductive characteristics, IR is regarded as the most common metabolic trait in PCOS patients (48). Numerous studies demonstrate that gut dysbiosis correlates with IR in PCOS (49, 50), and therapies aimed at the gut microbiota may mitigate IR (51). Dong et al. (26) found no significant disparity in the Shannon index between the total PCOS cohort and healthy controls. Subgroup analysis revealed that the Shannon index was markedly lower in the IR-PCOS group than in the non-IR PCOS group. This indicates that variations in α-diversity may be associated with particular phenotypes of PCOS. He et al. (23), in their study of individuals with IR-PCOS, identified an enrichment of the species *Rothia*, *Ruminococcus*, and *Enterococcus* in the gut microbiota. The prevalence of these bacterial groups exhibited a positive correlation with the HOMA-IR score and indicators of abdominal obesity, indicating their potential significance in the pathogenesis of the IR-PCOS phenotype.

## 3 Effects of gut microbiota metabolites on the metabolic and reproductive phenotypes of PCOS

### 3.1 SCFAs

**Figure 1:**
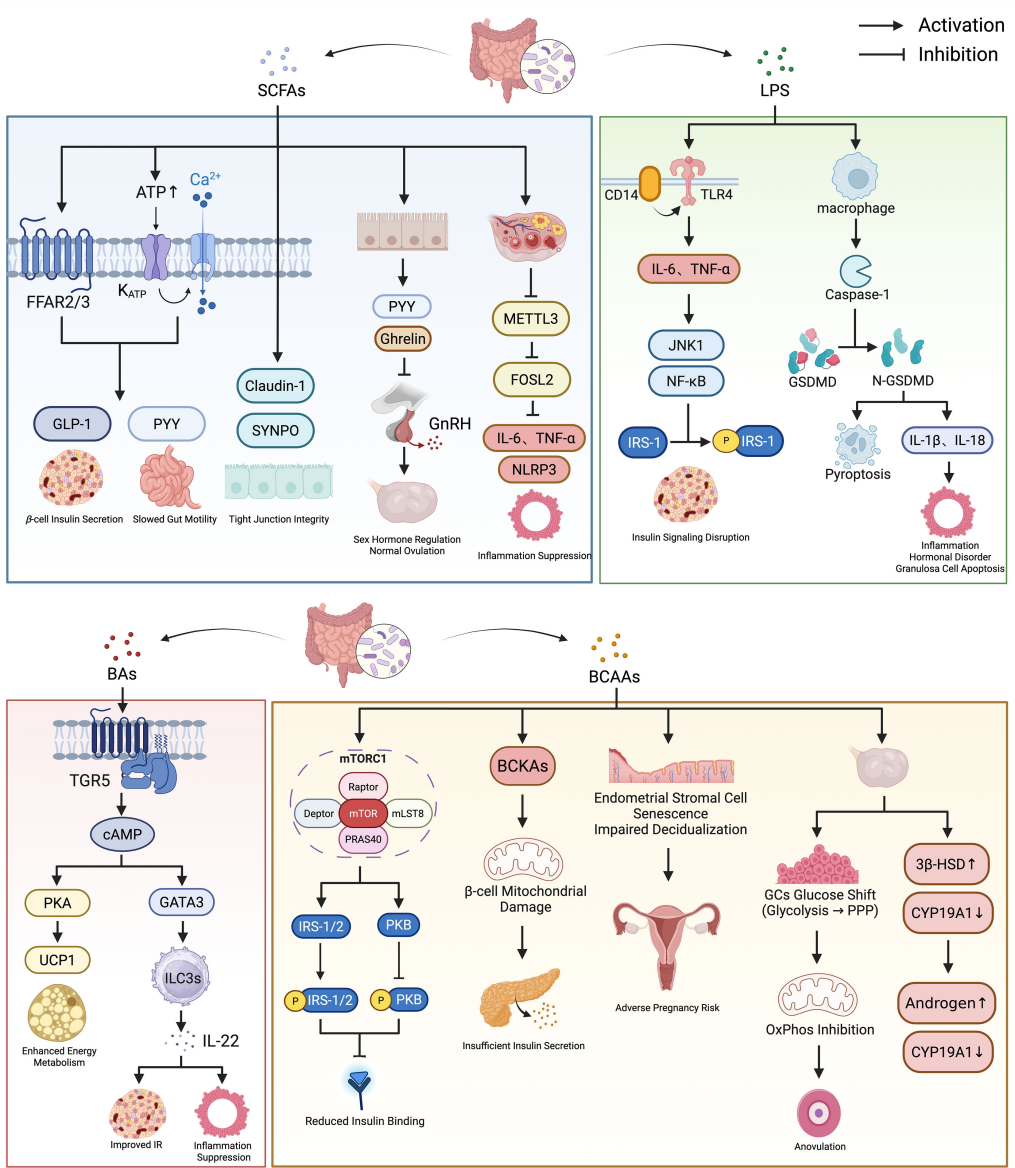
Effects of SCFAs, BAs, LPS, and BCAAs on the metabolic and reproductive phenotypes of PCOS.

SCFAs, metabolites produced by gut microbiota through fermenting dietary fiber, include acetate, propionate, butyrate, and valerate (52). A study by Zhang et al. (35) found that the intestinal content of acetate, propionate, and butyrate was reduced by approximately 30% to 66% in PCOS patients compared to healthy controls. Following 10 weeks of probiotic treatment in PCOS patients, the abundance of *Lactobacillus* in the gut significantly increased, accompanied by a consequent rise in SCFAs levels. Multiple studies have demonstrated a significant reduction in beneficial SCFAs-producing bacteria in PCOS patients, such as *Butyricicoccus*, *Blautia*, *Coprococcus*, *Faecalibacterium prausnitzii*, *the Clostridium innocuum group*, and *Prevotella*, leading to decreased intestinal SCFAs levels (53, 54). This dysbiosis disrupts the metabolic equilibrium between the host and the microbiota, thereby promoting the onset and progression of PCOS. (**Figure 1**).

#### 3.1.1 Effects of SCFAs on the metabolic phenotype of PCOS

SCFAs promote the intestinal secretion of peptide YY (PYY) and glucagon-like peptide-1 (GLP-1) by activating free fatty acid receptor 2 (FFAR2) and free fatty acid receptor 3 (FFAR3) (55). Specifically, GLP-1 stimulates insulin secretion from pancreatic β-cells, inhibits glucagon release, and delays gastric emptying, thereby helping to reduce postprandial blood glucose levels. PYY primarily reduces energy intake by suppressing appetite. Furthermore, it slows intestinal peristalsis, prolonging the time food spends in the gut for digestion and absorption, indirectly reducing the efficiency of energy uptake. Beyond the traditional FFAR2/FFAR3 pathway, in the colon, acetate and butyrate can be metabolized by colonic L-cells as energy substrates to generate adenosine triphosphate (ATP). Elevated ATP levels inhibit the ATP-sensitive potassium channels, leading to the depolarization of the L-cells’ membrane. This depolarization activates Ca^2+^ channels, promoting the influx of extracellular Ca^2+^, ultimately triggering calcium-dependent exocytosis and the release of GLP-1 and PYY (56). In addition, butyrate can promote the expression of claudin-1 and the actin-binding protein synaptopodin. This action strengthens the intercellular junction structure, regulates epithelial cytoskeletal stability, maintains tight junction integrity, reduces intestinal permeability, and consequently decreases the translocation of endotoxins (57).

#### 3.1.2 Effects of SCFAs on the reproductive phenotype of PCOS

Zhang et al. (35) observed that SCFAs deficiency in PCOS patients suppresses the secretion of gut-brain mediators by enteroendocrine cells, such as ghrelin and PYY. Abnormal levels of these mediators disrupt the signaling between the gastrointestinal tract and the central nervous system, leading to the disinhibition of the hypothalamus. This results in the excessive release of gonadotropin-releasing hormone (GnRH), which subsequently stimulates the pituitary to secrete luteinizing hormone (LH). Excessive LH acts on the ovaries, promoting the hypersecretion of T by theca cells, ultimately causing reproductive abnormalities in PCOS patients, such as anovulation and HA. Furthermore, Liu et al. (58) established a PCOS inflammatory model using Lipopolysaccharide (LPS)-induced human ovarian granulosa-like tumor cells (KGN). Their study demonstrated that butyrate inhibits the expression of the N6-methyladenosine (m6A) methyltransferase METTL3, thereby reducing the m6A modification level of its target gene, FOSL2. This reduction decreases FOSL2 mRNA stability and translation efficiency, ultimately lowering the levels of inflammatory cytokines, including interleukin-6 (IL-6) and tumor necrosis factor-α (TNF-α), and inhibiting the activation of the NOD-like receptor family pyrin domain containing 3 (NLRP3) inflammasome, thus suppressing inflammation and apoptosis in ovarian granulosa cells (GCs). Concurrently, *in vivo* studies using an obese PCOS mouse model further validated that butyrate supplementation improves ovarian morphology, suppresses ovarian inflammation, and regulates sex hormone levels, such as follicle-stimulating hormone (FSH), LH, and T.

### 3.2 BAs

BAs play a crucial role in the body through enterohepatic circulation. In the liver, cholesterol is utilized and catalyzed by the enzyme cholesterol 7α-hydroxylase (*CYP7A1*) to synthesize primary unconjugated BAs, which then conjugate with glycine or taurine to form primary conjugated BAs. These conjugated BAs are concentrated in the gallbladder and released into the intestine. The gut microbiota, including *Bacteroides vulgatus*, *Ruminococcus*, *Lachnospiraceae*, and *Prevotella*, employs BSH and 7α-dehydroxylase to deconjugate and dehydroxylate them, yielding secondary unconjugated BAs. These secondary unconjugated BAs are reabsorbed back into the liver, re-conjugated, and re-enter the intestine with bile, completing the circulation (12, 59). A study by Yu et al. (60) revealed alterations in the BAs profile of PCOS patients. Specifically, levels of primary bile acids, especially chenodeoxycholic acid (CDCA) and taurochenodeoxycholic acid (TCDCA), and of secondary unconjugated bile acids such as lithocholic acid (LCA) and deoxycholic acid (DCA) were significantly elevated, while the level of the secondary conjugated bile acid, glycodeoxycholic acid (GDCA), was decreased. (**Figure 1**).

#### 3.2.1 Effects of BAs on the metabolic phenotype of PCOS

BAs regulate glucose and lipid metabolism by activating signaling pathways such as the farnesoid X receptor (FXR) and takeda G protein-coupled receptor 5 (TGR5) (60). In brown adipose tissue (BAT) and muscle, TGR5 activation initiates adenylate cyclase signaling via Gαs protein-mediated mechanisms. This leads to increased intracellular cyclic adenosine monophosphate (cAMP) levels and subsequent activation of protein kinase A (PKA). Activated PKA then stimulates cAMP response element-binding protein and induces thyroid hormone deiodinase 2 (DIO2) expression. DIO2 catalyzes the conversion of inactive thyroxine to active triiodothyronine. This conversion enhances uncoupling protein 1 activity in BAT, promoting thermogenesis and increasing energy metabolism. Consequently, this pathway contributes to the reduction of obesity and fatty liver (61, 62). Regarding IR, Qi et al. (22) performed metagenomic sequencing on fecal samples from 50 PCOS patients and 43 healthy controls, revealing a significantly increased abundance of *Bacteroides vulgatus* in the gut microbiota of PCOS patients. This bacterium promotes the expression of BSH, which specifically cleaves the amide bond of conjugated BAs, such as GDCA, converting them into unconjugated BAs. The resulting decrease in GDCA levels inhibits the TGR5-cAMP pathway, downregulates the expression of the key transcription factor GATA binding protein 3 (GATA3), and subsequently reduces the secretion of interleukin-22 (IL-22) by intestinal group 3 innate lymphoid cells (ILC3s). The deficiency of IL-22 ultimately leads to IR.

#### 3.2.2 Effects of BAs on the reproductive phenotype of PCOS

The study by Qi et al. (22) demonstrated that the reduction of IL-22 resulting from the impaired TGR5-cAMP-GATA3 pathway leads to reproductive dysfunctions, including estrous cycle disturbances, altered ovarian morphology, abnormal hormone levels, and decreased first litter size. This detrimental effect is likely associated with the anti-inflammatory role of IL-22 within ovarian GCs. They showed that the administration of IL-22 reduces the secretion of CCL2, CCL20, and interleukin-1β (IL-1β) in GCs from individuals with PCOS. Specifically, CCL2 and CCL20 are responsible for recruiting immune cells to the ovarian locale, while IL-1β activates these immune cells and amplifies the inflammatory response, collectively disrupting the homeostasis of the ovarian microenvironment.

### 3.3 LPS

LPS, known as bacterial endotoxin, is a unique component of the cell wall of gram-negative bacteria (GNB) (63). Zheng et al. (29) showed in a study on female mice that serum LPS levels were significantly negatively correlated with the gut microbiota Chao1 index (r=-0.584, *p* = 0.007), suggesting that elevated LPS levels may be related to reduced gut microbiota richness. Notably, Guan et al. (64) found that the abundance of *Akkermansia muciniphila* in the intestines of PCOS model mice was significantly increased by 31.99% (*p* < 0.01) compared to the normal group. The overgrowth of *Akkermansia muciniphila* extensively consumes mucin in the intestinal mucus layer, leading to thinning of the mucus layer and a reduction in goblet cell numbers, thereby weakening the intestinal physical barrier. This impaired barrier not only induces local inflammation, but the metabolites produced by its mucin degradation also provide nutrients for other GNB, promoting their proliferation and increasing the total LPS synthesis (65). Dubey et al. (66) observed an elevated relative abundance of potential pathogens, including the phylum *Proteobacteria* and the family *Enterobacteriaceae*, in the gut microbiota of PCOS patients. These bacteria are capable of secreting toxic substances such as LPS and adhesins. Additionally, *Clostridium perfringens* can generate enterotoxins that compromise the tight junctions of the intestinal epithelium, thereby increasing intestinal permeability. This enhanced permeability facilitates the translocation of endotoxins into the systemic circulation, which activates a generalized immune response and triggers chronic low-grade inflammation (54). (**Figure 1**).

#### 3.3.1 Effects of LPS on the metabolic phenotype of PCOS

When LPS enters the bloodstream, it can bind to toll-like receptor 4 (TLR4) on the surface of immune cells via LPS-binding protein, cluster of differentiation 14 (CD14), and myeloid differentiation factor. This binding activates downstream signaling pathways and promotes the expression of inflammatory factors such as TNF-α and IL-6 (67, 68). These inflammatory factors can activate c-Jun N-terminal kinase 1 (JNK1) and Nuclear Factor kappa-light-chain-enhancer of activated B cells (NF-κB) signaling pathways, leading to the phosphorylation of serine residues on insulin receptor substrate-1 (IRS-1) protein, thereby hindering the normal binding of IRS-1 to the insulin receptor β-subunit, interrupting insulin signaling, and ultimately leading to IR (69).

#### 3.3.2 Effects of LPS on the reproductive phenotype of PCOS

Huang et al. (70) confirmed that elevated LPS in PCOS mice can reach the ovaries and be recognized by macrophages, activating intracellular caspase-11 to initiate a cascade reaction of pyroptosis, leading to the cleavage of the pyroptosis execution protein Gasdermin D (GSDMD) into its active N-terminal fragment (GSDMD-N). GSDMD-N further targets the macrophage membrane, forming large oligomeric pores, causing osmotic swelling and rupture of cells, and simultaneously releasing pro-inflammatory factors such as IL-1β and interleukin-18 (IL-18). These inflammatory factors not only exacerbate local inflammation but also affect GCs function through paracrine effects. On one hand, they inhibit the expression of 17β-hydroxysteroid dehydrogenase (17β-HSD) and estrogen receptor β (Esr2) in GCs, thereby hindering estrogen synthesis and signaling. On the other hand, they upregulate pro-apoptotic genes such as *Fas* and *Tnf* while downregulating anti-apoptotic genes such as *Bcl2* and *Bmp2*, leading to increased GCs apoptosis. Impaired GCs function and increased apoptosis result in estrogen synthesis disorders and follicular development arrest, ultimately manifesting as typical PCOS symptoms such as HA and polycystic ovarian morphology (PCOM).

### 3.4 Trimethylamine N-oxide

The production of TMAO is dependent on the gut microbiota’s metabolism of dietary metabolites. The gut microbiota metabolizes substances like choline, phosphatidylcholine, and L-carnitine to produce trimethylamine (TMA). After being absorbed into the bloodstream, TMA is further oxidized to TMAO by hepatic flavin-containing monooxygenases (FMOs) (71, 72). Specifically, mice studies have confirmed that *Lachnoclostridium saccharolyticum* and *Clostridium* sp*orogenes* can produce TMA through choline metabolism. Human metagenomic studies have shown that *γ-proteobacteria*, especially *Escherichia coli*, are the main TMA-producing bacteria (73, 74). It is known that TMAO is closely associated with various metabolic and cardiovascular diseases, including atherosclerosis, heart failure, IR, hepatic steatosis, and non-alcoholic fatty liver disease (NAFLD) (75–77). Due to gut microbiota dysbiosis being a key feature of PCOS, women with it are also at a higher risk of developing these related diseases.

#### 3.4.1 Effects of TMAO on the metabolic phenotype of PCOS

Our previous research (78) focuses on PCOS patients without HA. Analysis of 27 non-HA PCOS patients and 23 non-PCOS controls revealed that plasma TMAO levels were significantly elevated in non-HA PCOS patients (2.37 vs 1.65 μmol/L, *p* = 0.003), and this difference was more pronounced in the obese subgroup. Further analysis showed that in obese non-HA PCOS patients, plasma TMAO levels were positively correlated with the inflammatory cytokine interleukin-17A (IL-17A) (r=0.567, p<0.05). Furthermore, logistic regression analysis confirmed that TMAO is an independent predictor of non-HA PCOS (OR = 3.814, 95% CI: 1.330-10.939, *p* = 0.013). These results suggest that elevated TMAO may influence the pathogenesis of non-HA PCOS by participating in systemic inflammatory responses.

#### 3.4.2 Effects of TMAO on the reproductive phenotype of PCOS

A prospective study by Eyupoglu et al. (79) showed that TMAO levels in PCOS patients were significantly higher than those in healthy controls (2.39 vs 2.05 μmol/L, *p* = 0.042) and positively correlated with T (r=0.29, *p* = 0.037). After 3 months of intervention with oral contraceptives combined with a healthy lifestyle, the patients’ TMAO levels decreased from 3.35 to 2.05 (*p* = 0.002), along with reductions in T, free androgen index (FAI), and BMI, suggesting an association between elevated serum TMAO and HA. It is worth noting that there are differences between animal experiments and human studies. Our latest research (80), using a DHEA-induced PCOS mouse model, found that plasma TMAO levels were significantly reduced in PCOS mice, and TMAO supplementation enhanced mitochondrial function and oocyte quality, showing potential significance for improving assisted reproductive outcomes. Firstly, we speculate that the effect of TMAO may be bidirectional, meaning that both excessively high and low levels could have different impacts. In this study, the TMAO levels in PCOS mice were inherently low, and supplementing TMAO to restore them to the normal range might have exerted a protective effect. Secondly, this might be related to differences in TMAO regulatory pathways. For instance, DHEA-induced hyperandrogenic states in mice inhibit the expression of FMOs, leading to reduced TMAO production (81). In humans, however, TMAO levels are more influenced by gut microbiota composition and dietary precursors (82). Currently, the specific mechanism by which TMAO participates in PCOS is not fully elucidated, and its value as a potential biomarker for PCOS still requires further in-depth research to confirm (49).

### 3.5 BCAAs

BCAAs, including valine, leucine, and isoleucine, are essential amino acids that must be obtained through diet, as they cannot be synthesized endogenously by humans (83). They also serve as crucial substrates metabolized by the gut microbiota. Several studies indicate that BCAAs are significantly elevated in obese patients with PCOS, showing a positive correlation with both IR and HA (84). Paczkowska et al. (85) confirmed this observation, reporting significantly higher BCAAs in PCOS patients (540.59 ± 97.23 nmol/mL vs 501.09 ± 85.33 nmol/mL), particularly in those exhibiting HA. They further noted that BCAAs dysregulation was more pronounced in the subgroup of PCOS patients presenting with abdominal obesity. Multiple studies have identified *Prevotella copri* and *Bacteroides vulgatus* as key bacterial species involved in BCAAs synthesis within the human gut (40, 86). Thus, gut microbiota dysbiosis may contribute to PCOS pathology via the abnormal metabolism of BCAAs. Supporting this mechanism, animal experiments have demonstrated that colonization with *Prevotella copri* can elevate circulating BCAAs in mice, leading to the induction of IR and the exacerbation of glucose intolerance (40). A prospective cohort study conducted by Jing et al. (87) found that the abundance of *Parabacteroides merdae* was reduced in the gut of PCOS patients, and this reduction was associated with elevated serum levels of BCAAs, particularly isoleucine. (**Figure 1**).

#### 3.5.1 Effects of BCAAs on the metabolic phenotype of PCOS

Multiple potential mechanisms underlie the induction of IR by BCAAs. One primary mechanism involves the activation of the mammalian target of rapamycin complex 1 (mTORC1) by excessive BCAAs. mTORC1 activation subsequently leads to two critical events: the serine phosphorylation of insulin receptor substrates IRS-1 and insulin receptor substrate-2 (IRS-2) (88), and the inhibition of phosphorylation of protein kinase B (PKB), a key molecule in the insulin signaling cascade (89). These actions collectively impair the binding affinity between the insulin receptor and its substrates, thereby blocking insulin signal transduction and ultimately initiating or exacerbating IR. Furthermore, the central role of mTORC1 in BCAA-mediated insulin signaling suppression is validated by the finding that the mTORC1 inhibitor rapamycin can effectively reverse BCAAs-induced IR (90). An alternative mechanism suggests that the aberrant catabolism of BCAAs generates mitochondrially toxic metabolites, such as branched-chain α-keto acids (BCKAs) and branched-chain acyl-CoAs. These metabolites potentially impair pancreatic β-cell mitochondrial function. This mitochondrial dysfunction subsequently activates the c-Jun N-terminal Kinase (JNK)/p38 mitogen-activated protein kinase (p38) mitogen-activated protein kinase stress signaling pathway and triggers the release of cytochrome C, initiating the apoptotic cascade. The resulting failure of β-cell functional compensation and reduction in β-cell mass ultimately contributes to the development of type 2 diabetes mellitus (T2DM) (91).

#### 3.5.2 Effects of BCAAs on the reproductive phenotype of PCOS

Elevated isoleucine levels in the endometrium exacerbate the senescence of endometrial stromal cells, characterized by decreased cell proliferation capacity, enhanced senescence-associated β-galactosidase activity, cell cycle arrest, and increased levels of reactive oxygen species (ROS). Concurrently, this impairs the decidualization process, with the severity of damage correlating positively with increasing isoleucine concentration, ultimately increasing the risk of adverse pregnancy outcomes (87). Locally in the ovary, BCAAs accumulation drives an aberrant shift in glucose metabolism within ovarian GCs, promoting a transition from glycolysis towards the pentose phosphate pathway. This metabolic reprogramming is coupled with the inhibition of mitochondrial oxidative phosphorylation, thereby disrupting the energy homeostasis of the follicular microenvironment. Consequently, this leads to abnormal follicular development, manifesting as an increased number of preantral follicles and ovulatory dysfunction. Furthermore, BCAAs can induce HA by enhancing androgen synthesis and suppressing its conversion to estrogen. Mechanistically, BCAAs achieve this by upregulating 3β-hydroxysteroid dehydrogenase (3β-HSD), a key enzyme in androgen synthesis, and downregulating cytochrome P450 family 19 subfamily A member 1 (*CYP19A1*), the critical enzyme for androgen aromatization. This condition manifests clinically as elevated serum T and reduced levels of sex hormone-binding globulin (SHBG) (1).

### 3.6 Emerging gut metabolites

#### 3.6.1 3,4-Dihydroxyphenylacetic acid

Li et al. (92) reported significantly lower levels of DHPAA in the feces and serum of both PCOS patients and mouse models. DHPAA, a critical metabolite from the gut microbial degradation of dietary flavonoids, ameliorates PCOS by inhibiting the bone morphogenetic protein (BMP) signaling pathway. DHPAA specifically downregulates the expression of important genes in the ovarian BMP pathway, like *Bmp15* and *Gdf9*. Since *Bmp15* and *Gdf9* positively regulate the expression of anti-müllerian hormone (AMH), DHPAA is able to mitigate the abnormally elevated AMH levels associated with PCOS, thereby improving the PCOS reproductive phenotype. These improvements include reducing the number of ovarian cystic follicles, increasing the number of post-ovulatory corpora lutea, decreasing serum T levels, and restoring disrupted estrous cycles. Further investigation confirmed that the gut microbiota is an essential prerequisite for DHPAA production. *Streptococcus thermophilus* was recognized as a principal bacterium involved in DHPAA production by modulating β-galactosidase activity. This mechanism is supported by the observation that the relative abundance of *Streptococcus thermophilus* is significantly reduced in both PCOS patients and mouse models.

#### 3.6.2 Indole-3-propionic acid

Previous studies have shown that plasma tryptophan levels are abnormally elevated in PCOS patients (93). IPA, a key downstream product of tryptophan metabolism by intestinal microorganisms, has been shown to play an important role in metabolic diseases (94), and its synthesis is highly dependent on the homeostasis of the gut microbiota (95). Li et al. (96) systematically explored the association between IPA and PCOS and its potential mechanism of action. The results showed that the IPA levels in the feces and serum of PCOS patients and DHEA-induced PCOS model mice were significantly reduced. Supplementing IPA to PCOS model mice can activate the aryl hydrocarbon receptor (AhR), inhibit the activation of NLRP3 inflammasome and NF-κB pathway, and reduce the release of pro-inflammatory factors to alleviate ovarian inflammation. At the same time, it can improve the levels of antioxidant substances, reduce the oxidative damage marker and repair the mitochondrial function, so as to reduce oxidative stress. In addition, it can up-regulate the expression of thermogenesis-related genes in BAT and enhance fat thermogenesis. Finally, these mechanisms together led to the improvement of PCOS phenotypes such as IR, estrous cycle disorder, ovarian pathological damage, and hormone imbalance.

#### 3.6.3 Agmatine

Yun et al. (97) demonstrated that *Bacteroides vulgatus* in the gut metabolizes dietary arginine into agmatine through arginine decarboxylase (ADC), an enzyme encoded by its *speA* gene. Agmatine, acting as an agonist of intestinal FXR, activates the FXR signaling pathway in intestinal epithelial L cells, consequently inhibiting GLP-1 secretion. This reduction in GLP-1 levels induces IR and ovarian dysfunction, culminating in a PCOS-like phenotype in female mice. Importantly, this PCOS-like phenotype was ameliorated by either knocking out the *speA* gene in *Bacteroides vulgatus* or by administering the ADC inhibitor difluoromethylarginine (DFMA) to reduce agmatine production. Their prior work revealed that *Bacteroides vulgatus* can also induce PCOS-like phenotypes by down-regulating GDCA and TUDCA levels through the BSH pathway, which inhibits IL-22 secretion by ILC3s (22). The *speA*-dependent agmatine pathway, identified in the current study, represents an additional significant mechanism by which *Bacteroides vulgatus* contributes to PCOS, operating independently of BAs. These two distinct pathways can independently and synergistically induce PCOS-like phenotypes, and simultaneous targeting of both mechanisms was shown to more effectively ameliorate PCOS symptoms (97).

#### 3.6.4 AT-C1

Numerous studies have demonstrated that gut bacterial dysbiosis is closely associated with metabolic disorders and endocrine dysfunction in PCOS. However, mycobiota, another crucial component of the gut microbiota, possesses a more complex genome than bacteria and can produce a wider variety of secondary metabolites. The gut mycobiota has garnered significant attention in diseases such as metabolic dysfunction-associated steatohepatitis, inflammatory bowel disease (IBD), and gastrointestinal tumors (98–100), but its relationship with PCOS remains less explored. Wu et al. (101) investigated the structural differences in the gut mycobiota between PCOS patients and healthy controls. They discovered that the abundance of *Aspergillus tubingensis* was significantly higher in PCOS patients’ guts and that this fungus could stably colonize the gut. Animal experiments further demonstrated that *Aspergillus tubingensis* could induce PCOS-like phenotypes in mice by inhibiting the AhR-IL-22 pathway in ILC3s. These phenotypes included abnormal LH pulsatility, estrous cycle disruption, elevated T and LH levels, reduced ovarian corpus luteum count, aggravated IR, and decreased thermogenesis linked to lipogenesis. Using a screening procedure based on strain diversity and metabolite activity, the researchers subsequently identified AT-C1, a secondary metabolite of *Aspergillus tubingensis*. AT-C1 functions as an endogenous AhR antagonist, directly binding to AhR and potently suppressing its transcriptional activity, thereby reducing the secretion of IL-22. Clinically, fecal AT-C1 levels were significantly elevated in PCOS patients compared to healthy controls, showing a strong negative correlation with IL-22 and positive correlations with LH and T levels.

## 4 Interaction between metabolic and reproductive phenotypes

**Figure 2:**
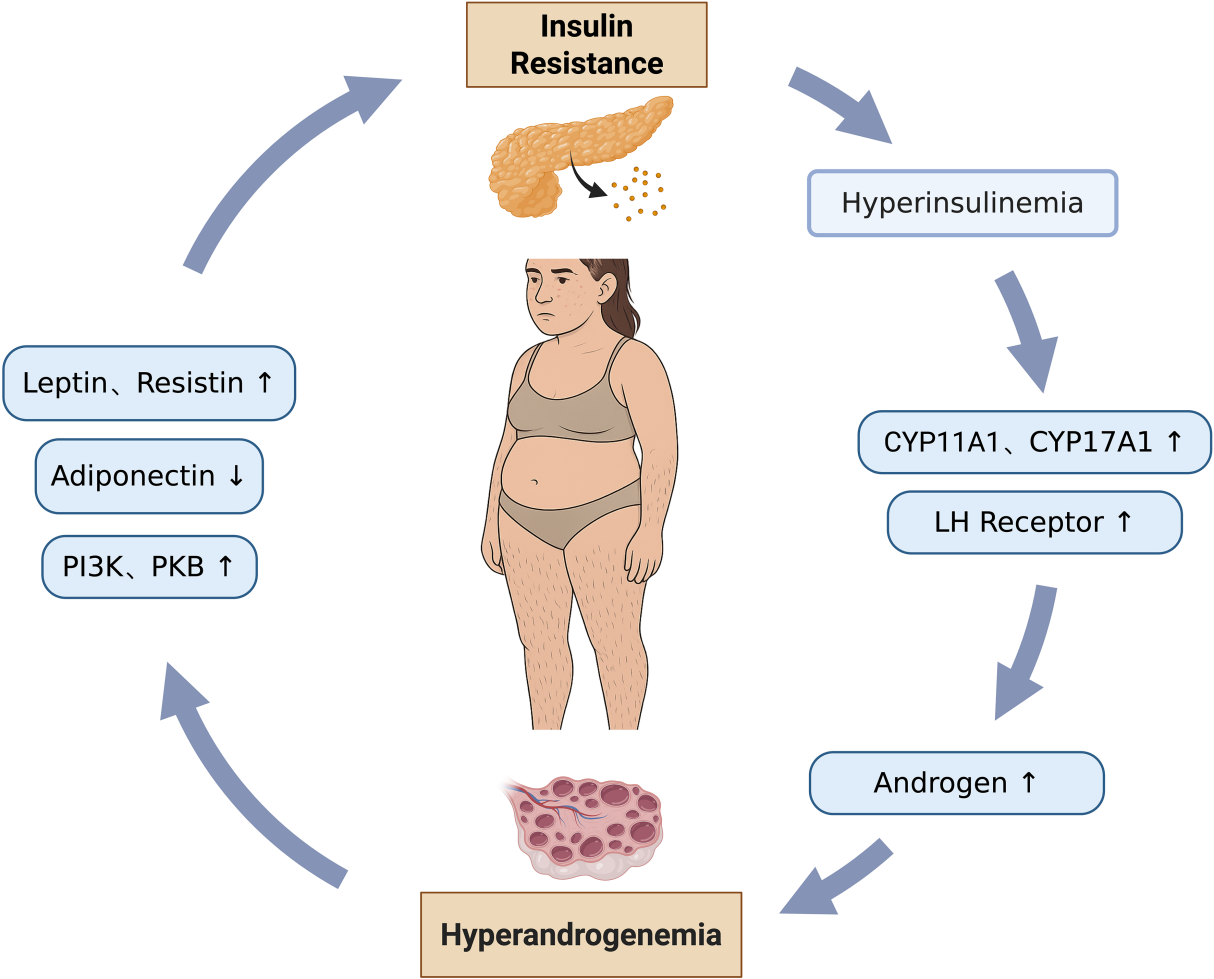
Interaction between metabolic and reproductive phenotypes.

In PCOS, the pathogenesis is characterized by a complex and bidirectional relationship between metabolic and reproductive phenotypes, where each dysfunction actively drives and exacerbates the other, forming a detrimental feedback loop. (**Figure 2**).

### 4.1 Metabolic phenotype driving reproductive dysfunction

The state of IR leads to hyperinsulinemia, where excessive insulin binds to its receptors on ovarian theca cells. This action upregulates the activity of key steroidogenic enzymes, specifically cytochrome P450 family 11 subfamily A member 1 (*CYP11A1*) and cytochrome P450 family 17 subfamily A member 1 (*CYP17A1*). *CYP11A1* catalyzes the conversion of cholesterol to pregnenolone, which serves as an androgen precursor, while *CYP17A1* further processes this precursor into biologically active androgens. This mechanism directly results in elevated ovarian androgen synthesis. Concurrently, insulin promotes the expression of LH receptors on the surface of theca cells, thereby amplifying the effects of androgen synthesis and exacerbating androgen oversecretion, culminating in HA (69, 102). The accumulation of excessive androgens within the follicles directly inhibits GCs proliferation and antrum formation, causing follicular arrest at the small antral follicle stage, thereby preventing their development into dominant follicles. Furthermore, patients with IR exhibit significantly elevated levels of AMH secreted by ovarian GCs. AMH inhibits follicular sensitivity to FSH, impeding the transition from primordial to preantral follicles. It also reduces aromatase activity, further suppressing estrogen synthesis, thereby aggravating follicular maturation defects and leading to the characteristic PCOM observed via ultrasound (48).

The endometrium is a target organ for insulin and its receptors, and its cells regulate glucose metabolism through surface glucose transporters (103, 104). Local IR in the endometrium can lead to insufficient glucose supply, interfering with endometrial growth and activity, reducing endometrial receptivity (ER), and thereby affecting embryo implantation and development (10, 105). This process is also closely related to inflammatory responses. PCOS patients have low-grade inflammation, which can interact with IR to promote each other (106). Inflammatory factors can activate the NF-κB signaling pathway, further producing more inflammatory factors such as TNF-α, IL-6, and IL-18 (107, 108). Among them, TNF-α can inhibit insulin receptor tyrosine phosphorylation, block insulin signaling pathways, and reduce the expression of endometrial glucose transporter 4 (GLUT-4), exacerbating local IR in the endometrium (109, 110).

### 4.2 Reproductive phenotype driving metabolic dysfunction

HA directly acts upon the cellular signaling pathways within insulin target organs, such as the liver, muscle, and adipose tissue, thereby inhibiting the normal physiological function of insulin. Within the insulin signaling cascade, androgens can suppress the phosphorylation and activation of key molecules, notably phosphatidylinositol 3-kinase (PI3K) and PKB. These molecules are central to insulin’s role in promoting glucose transport, glycogen synthesis, and lipogenesis. This blockage of signal transduction directly impedes the cell’s ability to effectively uptake and utilize glucose, consequently leading to IR (111, 112). In addition, HA can lower the levels of adiponectin, an insulin sensitivity-related adipokine and an important insulin sensitizer, whose reduction directly weakens the body’s responsiveness to insulin. Conversely, HA increases the release of pro-inflammatory adipokines, such as Leptin and Resistin. These factors not only exacerbate local chronic inflammation within adipose tissue but also act via systemic circulation on peripheral tissues, including the liver and skeletal muscle, further inhibiting the insulin signaling pathway and aggravating IR (113, 114). Adipocytes in PCOS patients are significantly larger than those in BMI-matched healthy women, and adipocyte size exhibits a positive correlation with the level of HA (115). These hypertrophied adipocytes demonstrate markedly reduced insulin sensitivity, coupled with decreased expression and content of GLUT-4. This results in impaired insulin-mediated glucose uptake capacity, directly inducing local IR within the adipose tissue (116).

## 5 Gut microbiota therapeutic approaches for PCOS

### 5.1 FMT

FMT aims to restore the healthy balance of the gut microbiota or treat gut-related diseases by transplanting fecal microbiota from a healthy donor into the patient’s intestine (117). Restoring a healthy gut microbiota through FMT can simultaneously improve both the metabolic and reproductive phenotypes of PCOS, positioning it as a potentially effective therapeutic strategy. Studies by Huang et al. (118) demonstrated that when fecal microbiota from PCOS patients were transplanted into germ-free mice, the recipient mice developed symptoms such as weight gain, IR, elevated T, abnormal estrous cycles, reduced corpus luteum count, and intestinal barrier damage. Concurrently, the abundance of specific gut bacteria, including *Faecalibaculum* and *Mediterraneibacter* genera, was found to be elevated in the gut. Martinelli et al. (119) observed that following the transplantation of fecal microbiota from healthy rats into PCOS model rats, all recipients showed restoration of normal estrous cycles, reduced androgen synthesis, normalization of ovarian morphology, and improved gut microbiota composition. Specifically, the abundance of *Lactobacillus* and *Clostridium* genera increased, while the abundance of the Prevotella genus decreased. Thus, FMT can be utilized both to establish PCOS animal models and potentially to modify PCOS-related phenotypes. Among current potential therapeutic strategies, FMT is considered the most holistic approach, as it encompasses the entire microbial ecosystem, including the bacteriome, virome, fungome, archaeome, and even the parasitome. However, research on FMT is predominantly based on animal models. Its efficacy and safety in humans require further validation and clarification, along with the need to explore optimal clinical implementation protocols.

### 5.2 Probiotics and prebiotics

Probiotics are active microorganisms in the gut that exert beneficial effects by maintaining microbial balance and regulating host immunity. Common genera include *Lactobacillus*, *Bacillus*, *Bifidobacterium*, *Streptococcus*, and *Enterococcus (*120). Prebiotics are organic substances that selectively promote the growth and metabolism of beneficial gut bacteria, such as galactooligosaccharides, fructooligosaccharides, inulin, and lactulose (121), and can improve the structure and function of the gut microbiota. Probiotics and prebiotics have a positive impact on the gut microecology, demonstrating significant efficacy in improving gut health, regulating immune responses, and assisting in the treatment of various diseases, thus offering new strategies and directions for biological therapy (122–124). Studies by Zhang et al. (35) indicate that dietary supplementation with *Bifidobacterium lactis V9* exerts regulatory effects on the gut microbiota and disease indices in PCOS patients. The probiotic, through effective colonization, significantly promotes the abundance of SCFAs-producing bacteria, such as *Faecalibacterium*, *Akkermansia*, and *Butyricimonas*, thereby directly elevating intestinal SCFAs levels. The increase in SCFAs levels, on one hand, lowers intestinal pH and enhances gut barrier integrity, and on the other hand, competitively inhibits the proliferation of LPS-producing pro-inflammatory bacteria, including *Parabacteroides*, *Clostridium*, and *Escherichia/Shigella*, consequently alleviating chronic low-grade inflammation mediated by LPS. Ultimately, this mechanism effectively reduces the LH/FSH ratio and T levels, and improves IR and dyslipidemia. Furthermore, prebiotics, such as inulin, have also shown positive effects. In a PCOS mouse model, inulin decreased the *Firmicutes*/*Bacteroidetes* ratio, increased the abundance of SCFAs-producing bacteria, and upregulated tight junction protein expression, enhancing intestinal barrier integrity. Simultaneously, inulin attenuated inflammation by inhibiting the TLR4/MyD88/NF-κB pathway and NLRP3 inflammasome in the ovaries. Based on the mechanism described above, inulin can improve glucose tolerance and insulin sensitivity, reduce the number of ovarian cystic follicles, increase the number of corpora lutea, and restore sex hormone levels and the estrous cycle (125).

### 5.3 Traditional Chinese medicine

TCM, encompassing Chinese herbal medicine, acupuncture, and dietary therapy, has a history of several centuries in China and shows significant potential in the treatment of PCOS, with its role in regulating gut microbiota gaining increasing attention. Recent studies indicate that certain TCM formulas may exert therapeutic effects by altering the composition of the gut microbiota. Therefore, identifying active compounds or formulas from Chinese herbs that target the gut microbiota for PCOS treatment has become a promising area of research (126, 127). Bailin (BL), a proprietary Chinese medicine composed of Cordyceps sinensis, has the effect of warming yang and tonifying qi. Research indicates that BL improves PCOS by modulating gut microbiota, specifically by reducing pro-inflammatory bacteria such as *Akkermansia* and increasing beneficial bacteria such as *Lactobacillus*. Furthermore, BL protects intestinal barrier integrity, reduces gut-derived LPS release, and activates the IRS1-PI3K-PKB insulin pathway to improve IR. Concurrently, it inhibits the LPS-TLR4 inflammatory pathway, thereby alleviating chronic inflammation (64). Zishen Qingre Lishi Huayu recipe (ZQLHR), a patented Chinese herbal formula, has also shown significant efficacy in PCOS treatment, effectively improving patients’ spontaneous ovulation rate and ovulatory menstruation rate. Its mechanism of action includes promoting the enrichment of butyrate-producing bacteria like *Lachnospira and Faecalibacterium*, and reducing pro-inflammatory bacteria such as *Escherichia-Shigella*, which collectively contribute to significant improvement in host metabolic function. FMT experiments have confirmed its ability to improve ovarian structure in PCOS mice and regulate serum concentrations of acetate, butyrate, and succinate (128). Additionally, acupuncture, as an ancient TCM therapy, can significantly regulate intestinal function and microbial composition via the gut-brain axis, thereby improving PCOS (129). Studies have found that electroacupuncture can increase the abundance of *Tenericutes* and *Prevotella_9*, improve the estrous cycle, sex hormone levels, and glucose tolerance in rats, reduce body weight and visceral and subcutaneous fat content, and increase BAT weight (130).

## 6 Discussion and future perspectives

Existing studies have confirmed that the gut microbiota and their metabolites participate in the pathogenesis and progression of PCOS by regulating hormone secretion and influencing metabolic pathways, suggesting a bidirectional interaction. However, PCOS exhibits high phenotypic heterogeneity. Current research often focuses on the overall PCOS population, lacking precise analysis of the composition, metabolomic profiles, and functional differences of the gut microbiota across distinct subtypes, such as HA, IR, or ovulatory dysfunction types. Future studies should leverage multi-omics technologies to identify core pathogenic microbial communities and key metabolite biomarkers specific to these different subtypes, thereby providing a basis for precise subtyping, diagnosis, and targeted intervention strategies for PCOS.

Furthermore, current research predominantly focuses on common bacterial metabolites, such as SCFAs, BAs, and LPS, while paying less attention to other microbial populations and their corresponding metabolites within the gut. Future efforts should intensify the exploration of novel metabolites, analyzing their distribution characteristics and dynamic changes in PCOS patients. Investigating their association with PCOS-related metabolic and reproductive abnormalities will help uncover potential metabolic substances involved in the pathological process of PCOS, thus enriching our understanding of the relationship between gut microbial metabolites and the disorder.

Moreover, although the influence of the gut microbiota and its metabolites on the metabolic and reproductive phenotypes of PCOS has been preliminarily revealed, the specific underlying mechanisms require deeper elucidation. Future research needs to further explore the interplay between the gut microbiota and host cellular signaling pathways, endocrine regulatory networks, and immune function, aiming to clarify the critical molecular pathways and functional nodes involved.

Finally, intervention strategies targeting the gut microbiota in PCOS, such as FMT, probiotics, and TCM, are largely confined to animal studies or small-scale clinical trials. There is a critical lack of large-scale, long-term follow-up randomized controlled trials to validate their efficacy and safety, and standardization of intervention protocols remains absent. Future directions necessitate conducting multi-center clinical studies to develop personalized intervention regimens, ultimately providing evidence to support the inclusion of gut microbiota-based therapies into clinical guidelines for PCOS treatment.

## 7 Conclusion

PCOS is a complex endocrine and metabolic disorder whose pathogenesis is increasingly understood to be crucially linked to the gut microbiota and its metabolites. This review delves into the pivotal role of gut microbiota dysbiosis in the occurrence and development of PCOS, highlighting that PCOS patients commonly exhibit alterations in gut microbial diversity and composition. Specifically, an increase in pro-inflammatory taxa and a reduction in beneficial bacteria significantly drive its core pathophysiological processes, such as HA, IR, and inflammation. Important gut microbiota metabolites, including SCFAs, BAs, LPS, TMAO, and BCAAs, serve as crucial mediators linking microbial activity to host metabolic and reproductive dysfunction. By modulating gut barrier function, inflammatory responses, and hormone synthesis, these metabolites collectively contribute to the intricate pathological network of PCOS. Consequently, intervention strategies targeting the gut microbiota, comprising FMT, probiotics, prebiotics, and TCM, have opened new avenues for PCOS treatment. These approaches demonstrate immense potential for restoring microbial balance and alleviating PCOS symptoms, promising more effective therapeutic options for patients.

## Glossary

PCOS: polycystic ovary syndrome
HA: hyperandrogenism
IR: insulin resistance
OTUs: operational taxonomic units
HOMA-IR: homeostasis model assessment of insulin resistance
BMI: body mass index
PD: phylogenetic diversity
ACE: abundance-based coverage estimator
ASVs: amplicon sequence variants
PCoA: principal coordinate analysis
PERMANOVA: permutational multivariate analysis of variance
SVs: sequence variants
PLS-DA: partial least squares discriminant analysis
ANOVA: analysis of variance
NMDS: non-metric multidimensional scaling
ANOSIM: analysis of similarities
CCA: canonical correspondence analysis
RDA: redundancy analysis
CAP: canonical analysis of principal coordinates
PCA: principal component analysis
DHT: dihydrotestosterone
DHEA: dehydroepiandrosterone
BAs: bile acids
BCAAs: branched chain amino acids
SCFAs: short chain fatty acids
BSH: bile salt hydrolase
FMT: fecal microbiota transplantation
T: testosterone
DHEAS: dehydroepiandrosterone sulfate
PYY: peptide YY
GLP-1: glucagon-like peptide-1
FFAR2: free fatty acid receptor 2
FFAR3: free fatty acid receptor 3
ATP: adenosine triphosphate
GnRH: gonadotropin-releasing hormone
LH: luteinizing hormone
LPS: lipopolysaccharide
m6A: N6-methyladenosine
IL-6: interleukin-6
TNF-α: tumor necrosis factor-α
NLRP3: NOD-like receptor family pyrin domain containing 3
GCs: granulosa cells
FSH: follicle-stimulating hormone
CYP7A1: cholesterol 7α-hydroxylase
CDCA: chenodeoxycholic acid
TCDCA: taurochenodeoxycholic acid
LCA: lithocholic acid
DCA: deoxycholic acid
GDCA: glycodeoxycholic acid
FXR: farnesoid X receptor
TGR5: takeda G protein-coupled receptor 5
BAT: brown adipose tissue
cAMP: cyclic adenosine monophosphate
PKA: protein kinase A
DIO2: deiodinase 2
GATA3: GATA binding protein 3
IL-22: interleukin-22
ILC3s: group 3 innate lymphoid cells
IL-1β: interleukin-1β
GNB: gram-negative bacteria
TLR4: toll-like receptor 4
CD14: cluster of differentiation 14
JNK1: c-Jun N-terminal kinase 1
NF-κB: nuclear factor kappa-light-chain-enhancer of activated B cells
IRS-1: insulin receptor substrate-1
IL-18: interleukin-18
17β-HSD: 17β-hydroxysteroid dehydrogenase
Esr2: estrogen receptor β
PCOM: polycystic ovarian morphology
TMAO: trimethylamine N-oxide
TMA: trimethylamine
FMOs: flavin-containing monooxygenases
NAFLD: non-alcoholic fatty liver disease
IL-17A: interleukin-17A
FAI: free androgen index
mTORC1: mammalian target of rapamycin complex 1
IRS-2: insulin receptor substrate-2
PKB: protein kinase B
BCKAs: branched-chain α-keto acids
JNK: c-Jun N-terminal kinase
p38: p38 mitogen-activated protein kinase
T2DM: type 2 diabetes mellitus
ROS: reactive oxygen species
3β-HSD: 3β-hydroxysteroid dehydrogenase
CYP19A1: cytochrome P450 family 19 subfamily A member 1
SHBG: sex hormone-binding globulin
DHPAA: 3,4-dihydroxyphenylacetic acid
BMP: bone morphogenetic protein
AMH: anti-müllerian hormone
IPA: indole-3-propionic acid
AhR: aryl hydrocarbon receptor
ADC: arginine decarboxylase
DFMA: difluoromethylarginine
IBD: inflammatory bowel disease
CYP11A1: cytochrome P450 family 11 subfamily A member 1
CYP17A1: cytochrome P450 family 17 subfamily A member 1
ER: endometrial receptivity
GLUT-4: glucose transporter 4
PI3K: phosphatidylinositol 3-kinase
TCM: traditional Chinese medicine
BL: bailin
ZQLHR: Zishen Qingre Lishi Huayu recipe.
